# 

**Published:** 1893-11

**Authors:** 


### CONTENTS.

COMMUNICATIONS.

The Widening Field of the Dental Profession ........................ 521

PROCEEDINGS.

World's Columbian Dental Congress—1893 .......... .................... 531

Ohio State Dental Society—Notice ........................................ 586

EDITORIAL.

Ohio State Dental Society ................................................ 569

Correction, ................................................................ 570

In Memory of Dr. Lardlaw ..... ............................................ 571

Obituary—Dr. Geo. W. McElhaney ..... .................... ... ...... 572

